# Association of pulsatility index with total burden of cerebral small vessel disease and cognitive impairment

**DOI:** 10.1002/brb3.3526

**Published:** 2024-05-23

**Authors:** Huijuan Wu, Liaoyang Xu, Xingyongpei Zheng, Caihong Gu, Xinyu Zhou, Yong Sun, Xiaomin Li

**Affiliations:** ^1^ Department of Neurology Jinzhou Medical University Jinzhou China; ^2^ Department of Neurology Lianyungang Clinical College of Nanjing Medical University, The First People's Hospital of Lianyungang City Lianyungang China; ^3^ Department of Neurology The First Affiliated Hospital of Kangda College of Nanjing Medical University Lianyungang China; ^4^ Department of Neurology The Affiliated Lianyungang Hospital of Xuzhou Medical University Lianyungang China; ^5^ Department of Critical Care Medicine The First Affiliated Hospital of Kangda College of Nanjing Medical University Lianyungang China; ^6^ Department of Neurosurgery The Affiliated Lianyungang Hospital of Xuzhou Medical University The First People's Hospital of Lianyungang City Lianyungang China; ^7^ Department of Emergency Medicine Lianyungang Clinical College of Nanjing Medical University The First People's Hospital of Lianyungang Lianyungang China

**Keywords:** cerebral small vessel disease, cognitive impairment, pulsatility index, total burden

## Abstract

**Objective:**

This study investigated the correlation between the pulsatility index (PI) of the middle cerebral artery with the total burden of cerebral small vessel disease and cognitive impairment.

**Method:**

Information on patients hospitalized in the Department of Neurology was collected retrospectively. These patients had complete clinical and laboratory data. The middle cerebral artery PI was measured using transcranial Doppler, a Mini‐Mental State Examination (MMSE) was used to assess cognitive function, and the total cerebral small vessel disease burden was assessed using magnetic resonance imaging. Patients were grouped according to their scores for total imaging burden of cerebral small vessel disease and cognitive function. Logistic regression analysis assessed the association between the PI, total imaging burden, and cognitive impairment. Spearman analysis was used to evaluate the correlation between the PI and total imaging burden and cognitive impairment, and receiver operating characteristic (ROC) curves were used to determine the predictive value of the PI for cognitive function.

**Results:**

The PI was higher in the cognitive impairment (CI) group than in the no‐CI group. Binary logistic regression analysis showed that increased PI was an independent risk factor for CI (OR = 1.582; 95% CI: 1.043–2.401; *p* = .031) and total imaging burden (OR = 1.842; 95% CI: 1.274–2.663; *p *= .001). Spearman analysis found that the PI correlated negatively with the MMSE score (*r *= −.627, *p* < .001). ROC curve analysis showed the PI predicted CI with an area under the curve of 0.784. The PI combined with the total imaging burden predicted CI in cerebral small vessel disease with an area under the curve of 0.832.

**Conclusion:**

An increased PI was associated with CI and a high imaging burden in cerebral small vessel disease patients. The PI combined with the total burden score shows a high predictive value for CI.

## Graphical abstract

This study investigated the middle cerebral artery pulsatility index association with small vessel disease load and cognitive impairment. The pulsatility index was negatively correlated with cognitive function and positively correlated with total imaging burden. The middle cerebral artery pulsatility index and the total burden score show a high predictive value for cognitive decline.

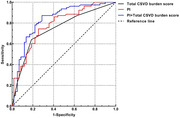

## 1 INTRODUCTION

Cerebral small vessel disease (CSVD) encompasses several clinical, pathological, and imaging conditions that arise from diverse etiologies affecting small arteries, micro‐arterioles, capillaries, venules, and small veins in the brain (Wardlaw et al., 2019). CSVD is primarily observed in imaging as lacunar infarcts (LI), white matter hyperintensities (WMH), enlarged perivascular spaces (EPVS), and cerebral microbleeds (CMBs) (Li et al., 2018). CSVD is known to be a leading cause of cognitive impairment (CI) (Hamilton et al., 2021), thereby imposing a significant burden and presenting a substantial challenge to public health and society on a global scale (Bos et al., 2018; Debette et al., 2019). The pathophysiological mechanisms underlying CSVD are still to be fully elucidated. It may be related to blood–brain barrier dysfunction, impaired vascular reactivity, vascular sclerosis, damage to the vessel wall, increased pulsatility, impairment of interstitial fluid drainage, and an enlarged perivascular gap (Wardlaw et al., 2019; Wardlaw et al., 2013). There is evidence that the formation of CSVD is intimately associated with increased intracranial vascular pulsatility (Webb et al., 2012), mainly associated with intracranial vascular sclerosis and increased resistance (Shi et al., 2018; Webb et al., 2012).

Transcranial Doppler ultrasound (TCD) is used to assess the middle cerebral artery pulsatility index (PI). This reflects distal small vessel resistance and the status of cerebral blood flow perfusion, which is related to intracranial arteriosclerosis and increased small vessel resistance (Vinciguerra et al., 2019; Wagshul et al., 2011). In CSVD patients, middle cerebral artery PI assessment with TCD is a feasible predictor of the occurrence and severity of future dementia risk (Fu et al., 2019; Vinciguerra et al., 2019). Previous research has indicated that an elevated PI in the middle cerebral artery correlates with various imaging markers and declining cognitive function in CSVD (Altmann et al., 2016; Kneihsl et al., 2020; Lau et al., 2018; Mok et al., 2012). However, these studies are mostly limited to single imaging markers. Various imaging markers are interrelated and exist simultaneously. The total burden of CSVD links various imaging markers together, which can better reflect the severity of CSVD damage (Staals et al., 2014; Wardlaw et al., 2013).

The present study explores the relationship between middle cerebral artery PI and CSVD total burden and cognitive dysfunction. It also analyzes the predictive value of PI combined with the total burden of cognitive dysfunction.

## 2 PATIENTS AND METHODS

### 2.1 Study cohort

This is a retrospective of CSVD patients hospitalized in the Department of Neurology from September 2021 to June 2023. A total of 137 individuals were included in this study; 92 were male (67.2%) and 45 were female (32.8%).This sthdy was approved by the ethics committees of the First People's Hospital of Lianyungang (KY‐20230612001‐01)

### 2.2 Inclusion criteria

(1) All enrolled patients were 55–85 years old. (2) All patients underwent MRI examination; complete MRI sequences were completed; and the total burden score was calculated. (3) All selected patients have complete demographic characteristics, general clinical data, biochemical indicators, and various neuroimaging examinations such as cranial MRI and TCD ultrasound. (4) They were able to cooperate in completing MMSE psychological assessment. (5) They agreed to participate in this research.

### 2.3 Exclusion criteria

(1) Patients with occlusion or moderate‐to‐severe stenosis of the carotid artery, vertebral artery, and subclavian artery detected by arterial ultrasound; (2) concomitant serious internal medicine diseases, such as atrial fibrillation or severe arrhythmia, cardiogenic embolism, and heart, lung, liver, and kidney dysfunction; (3) clearly diagnosed neurodegenerative diseases, degenerative diseases of the nervous system, hereditary CSVD, or amyloid angiopathy in the brain; (4) patients with visual or hearing dysfunction that significantly affect neuropsychological testing; (5) suffering from other brain or systemic disease that could affect cognitive function (e.g., Alzheimer's disease or other types of dementia, neurodemyelinating disorders, mental disorders, emotional disorders or multiple organ dysfunction, malignant tumors, etc.); (6) patients who take cognitive improvement, antipsychotic, and antidepressant drugs; (7) other special causes of brain white matter changes (such as multiple sclerosis, optic neuromyelitis, and brain white matter malnutrition); (8) patients with missing or poorly displayed temporal windows;(9) TCD examination having detected a significant increase in blood flow velocity and abnormal blood flow spectral pattern; (10) patients with incomplete required data results.

### 2.4 Methods

#### 2.4.1 Clinical data

All the included patients were collected for age, sex, drinking and smoking history, body mass index, education level, previous hypertension, diabetes history, systolic blood pressure (BP), diastolic BP, and pulse pressure difference. Laboratory data including high‐density lipoprotein (HDL), low‐density lipoprotein (LDL), triglyceride, total cholesterol, homocysteine, and renal function (creatinine and cystatin C) were collected.

#### 2.4.2 TCD exam

All subjects completed the TCD ultrasound examination within 7 days of admission. An experienced physician performed this examination using TCD sonography (Multi‐Dop digital, Compumedics DWL, Singen, Germany). The signal‐to‐noise ratio of this instrument is 63. The patient lies flat and remains calm. A 2MHZ pulse probe is placed in the temporal window of the patient to detect bilateral middle cerebral arteries M1 segment at a depth of 50–65 mm; if the optimum waveform cannot be obtained, the depth probe with the best waveform is selected. The physician performed angle corrections during the examination, and all waveforms were visually inspected to exclude interference. Recorded TCD ultrasound parameters, including peak systolic velocity (*V*_s_), end‐diastolic velocity (*V*_d_), mean flow velocity (*V*_m_), and PI. Average values of *V*_s_, *V*_d_, *V*_m_, and PI for bilateral middle cerebral arteries were selected as the analysis data for this study, where PI = (*V*_s_−*V*_d_)/*V*_m_.

#### 2.4.3 Imaging‐related indicators

Patients underwent cranial MRI evaluation with 3.0 tesla MRI scanners (Signa HDx, GE Healthcare), including T1‐weighted imaging (T1W1), T2‐weighted imaging (T2W2), fluid attenuation imaging (FLAIR), magnetic sensitivity weighted imaging (SWI), and diffusion‐weighted imaging (DWI).

The score for total burden was based on the imaging characteristics of CSVD patients (Staals et al., 2014), including asymptomatic LI, extensive WMH, deep CMBs, and EPVS. CSVD imaging markers were defined according to the Standards for Reporting Vascular Changes in Neuroimaging (STRIVE) (Wardlaw et al., 2013). The total burden was scored by two independent neuroimaging physicians, and conflicting results were evaluated by a third physician. (1) Lacunar infarction score (Wardlaw et al., 2013): round or ovoid subcortical lesions with signal similar to cerebrospinal fluid, low signal at T1WI, high signal at T2WI, and a lesion size of 3–15 mm. (2) WMH score (Fazekas et al., 1993): Based on T2WI or FLAIR imaging showing high signal intensity in paraventricular or deep white matter, the severity of WMH was evaluated on the Fazekas grading standard, and was rated as 1 when the paraventricular WMH signal intensity is 3 points, or when the deep WMH signal intensity is ≥ 2 points, it is rated as 1 point. (3) CMBs score (Gregoire et al., 2009): A signal loss lesion with a small or oval shape, diameter < 10 mm and clear boundary in the SWI sequence is rated 1 point. (4) EPVS score (Doubal et al., 2010): Circular or linear cerebrospinal fluid with high signal along the surrounding blood vessels, visible on T2WI with a diameter of < 3 mm, and no high signal surround on FLAIR sequence, using a visual quantitative scoring scale for scoring: 0, no EPVS; 1, 1–10 EPVS; 2, 11–20 EPVS; 3, 21–40 EPVS; 4, > 40 EPVS. When the basal ganglia score is 2, 3, or 4, it is 1 point. Calculate the sum of imaging marker scores, which is the total burden of CSVD imaging. The low‐burden group was assigned a score of 1–2, and the high‐burden group a score of 3–4.

#### 2.4.4 Assessment of cognitive function

Eligible patients underwent cognitive function assessment using a Chinese adaptation of the Mini‐Mental State Examination (MMSE). Due to varying education levels received by patients, different levels of education were considered when grouping cognitive dysfunction. According to previous studies, the optimal cut‐off scores for CI in the Chinese population were limited to 0–17 for illiterate, 0–20 for 1–6 years of education, and 0–24 for more than 7 years of education (Li et al., 2016).

### 2.5 Statistical analyses

Data analysis was conducted with SPSS 26.0. Numerical data are presented as the mean ± SD for normally distributed variables, and a comparison between groups was conducted with the Student's *t*‐test. Non‐normally distributed data were presented as the median (interquartile range), and groups were compared with the Mann–Whitney *U* test. Categorical data are shown as a frequency or percentage, and group comparisons using the *x^2^* test. Binary logistic regression was used to examine associations between PI and cognitive function or total burden of CSVD. Significant differences were considered to be *p* < .05. Relationships between PI, CSVD total burden, and cognitive function were examined using Spearman correlation analysis. Receiver operating characteristic (ROC) curves were employed to estimate predictive values of PI and CSVD burden for CI.

## 3 RESULTS

### 3.1 Patient characteristics

This study included 137 patients. The mean age of the participants was 68.80 ± 6.79 years; 92 were male (67.2%). These were categorized into two groups according to the MMSE score and education level: no‐CI group 59 cases, MMSE: 27.00 (25.00–28.00); and CI group 78 cases, MMSE: 19.00(17.00–22.00). The basic characteristics of the two groups are shown in Table 1. The patients in the CI group were older and had a higher history of hypertension than those in the group no‐CI; this difference was statistically significant (*p *< .05). In addition, compared to the no‐CI group, the patients in the CI group had higher PI levels, triglycerides, pulse pressure difference, cystatin C, total burden score, and lower HDL levels and education levels (*p *< .05).

**TABLE 1:** General characteristics of no cognitive impairment and cognitive impairment groups.

Variables	No cognitive impairment (*n *= 59)	Cognitive impairment group (*n *= 78)	*t/Z/χ^2^*	*p*
Sex (male), *n* (%)	42 (71.2)	50 (64.1)	0.764	.382
Age, mean (SD), years	65.08 ± 6.23	71.60 ± 5.79	6.311	<.001
Smoking history, *n* (%)	23 (39.0)	40 (51.3)	2.046	.153
Drinking history, *n* (%)	19 (32.2)	25 (32.1)	0.001	.985
Hypertension, (%)	24 (40.7)	49 (62.8)	6.616	.010
Diabetes mellitus, (%)	13 (22.0)	21 (26.9)	0.430	.512
Education level, median (IQR), year	8.00 (7.00, 11.00)	7.00 (4.00, 8.00)	−4.658	<.001
SBP on admission, mean (SD), mmHg	139.73 ± 17.17	142.40 ± 16.63	0.917	.361
DBP on admission, mean (SD), mmHg	87.85 ± 9.86	84.53 ± 11.30	1.799	.074
Pulse pressure, median (IQR), mmHg	52.00 (43.00, 57.00)	55.50 (50.75, 63.25)	−2.769	.006
TC, mean (SD), mmol/L	4.05 ± 1.11	4.11 ± 1.04	0.324	.747
Triglyceride, median (IQR), mmol/L	1.13 (0.86, 1.54)	1.46 (1.00, 1.86)	−2.067	.039
HDL‐C, mean (SD), mmol/L	1.31 ± 0.32	1.05 ± 0.21	5.549	<.001
LDL‐C, median (IQR), mmol/L	2.39 (1.84, 3.14)	2.38 (1.84, 3.20)	−0.406	.684
BUN, median (IQR), mmol/L	5.91 (4.99, 7.20)	5.86 (4.84, 7.36)	−0.072	.943
Creatinine, median (IQR), µmol/L	69.10 (56.40, 78.50)	62.75 (53.55, 72.98)	−1.621	.105
Cys C, median (IQR), mg/L	0.84 (0.74, 1.00)	0.99 (0.84, 1.16)	−3.213	.001
Hcy, median (IQR), µmol/L	16.00 (12.80, 18.20)	16.50 (13.88, 20.93)	−1.763	.078
PI	0.90 ± 0.13	1.04 ± 0.15	5.708	<.001
Total burden score	2.00 (1.00, 2.00)	3.00 (2.00, 3.00)	−5.141	<.001

Abbreviations: SBP, systolic BP; DBP, diastolic BP; TC, total cholesterol; HDL‐C, high‐density lipoprotein; LDL‐L, low‐density lipoprotein; BUN, blood urea nitrogen; Cys C, cystatin C; Hcy, Homocysteine; PI, pulsatility index.

### 3.2 Pulsatility index and cognitive impairment

**FIGURE 1:**
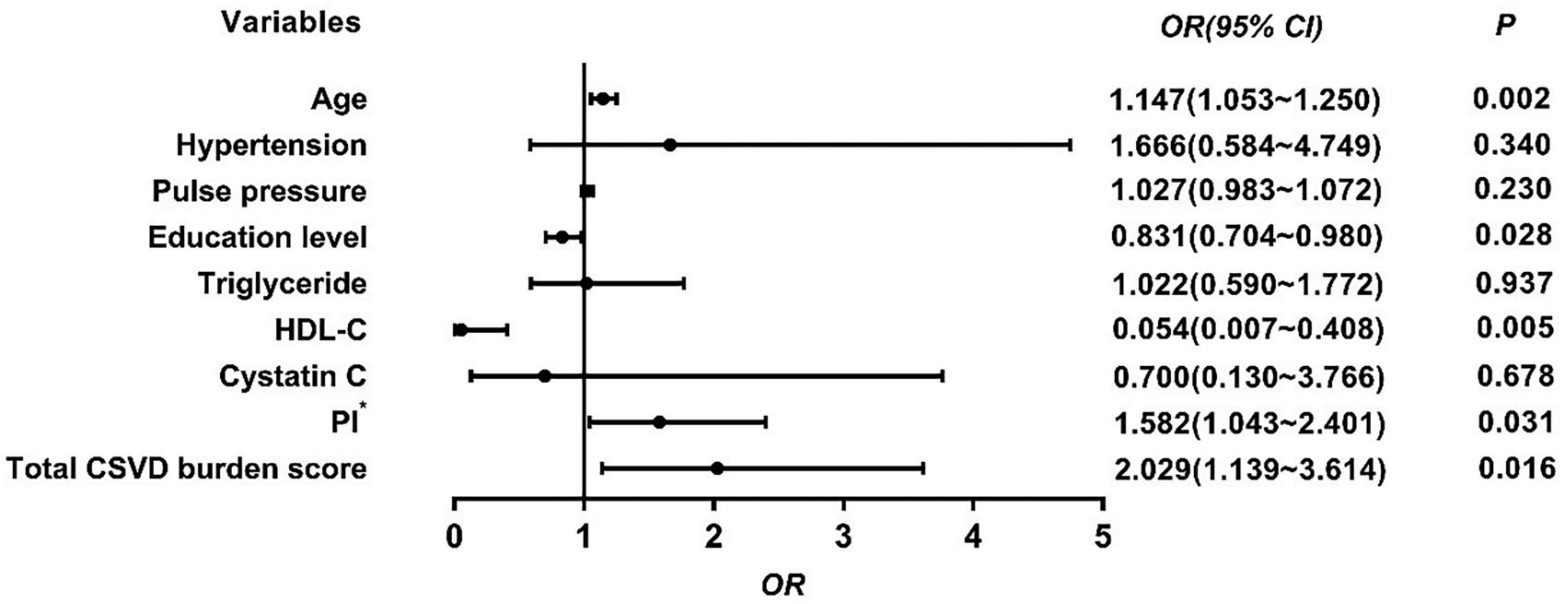
Binary logistic regression analysis for cognitive impairment of risk factors. TG, triglyceride; HDL‐C, high‐density lipoprotein; PI, pulsatility index. *, PI was multiplied by 10 and incorporated into the model.

The PI level in the CI group was significantly higher (*p* < .001) than in the no‐CI group (Table 1). After adjusting for age, education, hypertension, HDL, triglycerides, cystatin C, and burden of severe CSVD, multivariate binary logistic regression analysis found that this correlation remained significant (OR = 1.582, 95% CI: 1.043–2.401, *p *= .031), and that PI was an independent risk factor for cognitive impairment in CSVD patients (Figure 1). Further study revealed that PI and cognitive function were negatively correlated (*r *= −.627, *p* < .001, Table 2).

**TABLE 2:** Correlation between pulsatility index (PI) and total burden and Mini‐Mental State Examination (MMSE) score.

Variables	MMSE score	
	*R*	*P*
PI	−.627	<.001
Total burden score	−.712	<.001

### 3.3 Pulsatility index and high CSVD burden

Based on their total CSVD burden, patients were divided into low‐burden and high‐burden groups (Table 3). The high‐burden group exhibited a higher average age, greater prevalence of smoking and hypertension, and lower education level, triglycerides, high‐density lipoprotein, cystatin C, and PI compared to the low‐burden group (*p *< .05).

**TABLE 3:** Characteristics of participants between high burden and low burden.

Variables	High burden (*n *= 76)	Low burden (*n *= 61)	*t/Z/χ^2^*	*P*
Sex (male), *n* (%)	55 (72.4)	37 (60.7)	2.105	.147
Age, mean (SD), years	66.91 ± 6.59	71.15 ± 6.32	3.810	<.001
Smoking history (%)	29 (38.2)	34 (55.7)	4.210	.040
Drinking history (%)	27 (35.5)	17 (27.9)	0.910	.340
Hypertension (%)	31 (40.8)	42 (68.9)	10.706	.001
Diabetes mellitus (%)	22 (28.9)	12 (19.7)	1.560	.212
SBP on admission, mean (SD), mmHg	140.26 ± 16.43	142.48 ± 17.43	0.762	.447
DBP on admission, mean (SD), mmHg	86.33 ± 10.59	85.49 ± 11.10	0.450	.653
Pulse pressure, median (IQR), mmHg	54.5 (44.75, 58.75)	54 (49, 63.5)	‐1.463	.144
Education level, median (IQR), year	8 (7.0, 10.75)	6 (3.5, 8.0)	‐4.087	<0.001
TC, mean (SD), mmol/L	4.14 ± 1.16	4.02 ± 0.95	0.637	.525
Triglyceride, median (IQR), mmol/L	1.07 (0.86, 1.62)	1.47 (1.14, 1.93)	−3.292	.001
HDL‐C, mean (SD), mmol/L	1.21 ± 0.30	1.11 ± 0.27	2.007	.047
LDL‐C, median (IQR), mmol/L	2.45 (1.86, 3.28)	2.35 (1.81, 3.10)	−1.055	.292
BUN, median (IQR), mmol/L	5.73 (4.88, 6.49)	6.01 (4.87, 7.58)	−1.005	.315
Creatinine, median (IQR), µmol/L	67.8 (56, 75.9)	63.3 (53.5, 77.5)	−0.669	.503
Cys C, median (IQR), mg/L	0.84 (0.74, 0.97)	1.11 (0.91, 1.18)	−5.179	<.001
Hcy, median (IQR), µmol/L	15.95 (13.13, 18.43)	16.7 (13.85, 20.1)	−1.321	.186
PI	0.91 ± 0.14	1.06 ± 0.14	6.340	<.001

Abbreviations: SBP, systolic BP; DBP, diastolic BP; TC, total cholesterol; HDL‐C, high‐density lipoprotein; LDL‐L, low‐density lipoprotein; BUN, blood urea nitrogen; Hcy, homocysteine; PI, pulsatility index.

### 3.4 Multivariate logistic regression analysis of PI and CSVD total burden

**FIGURE 2:**
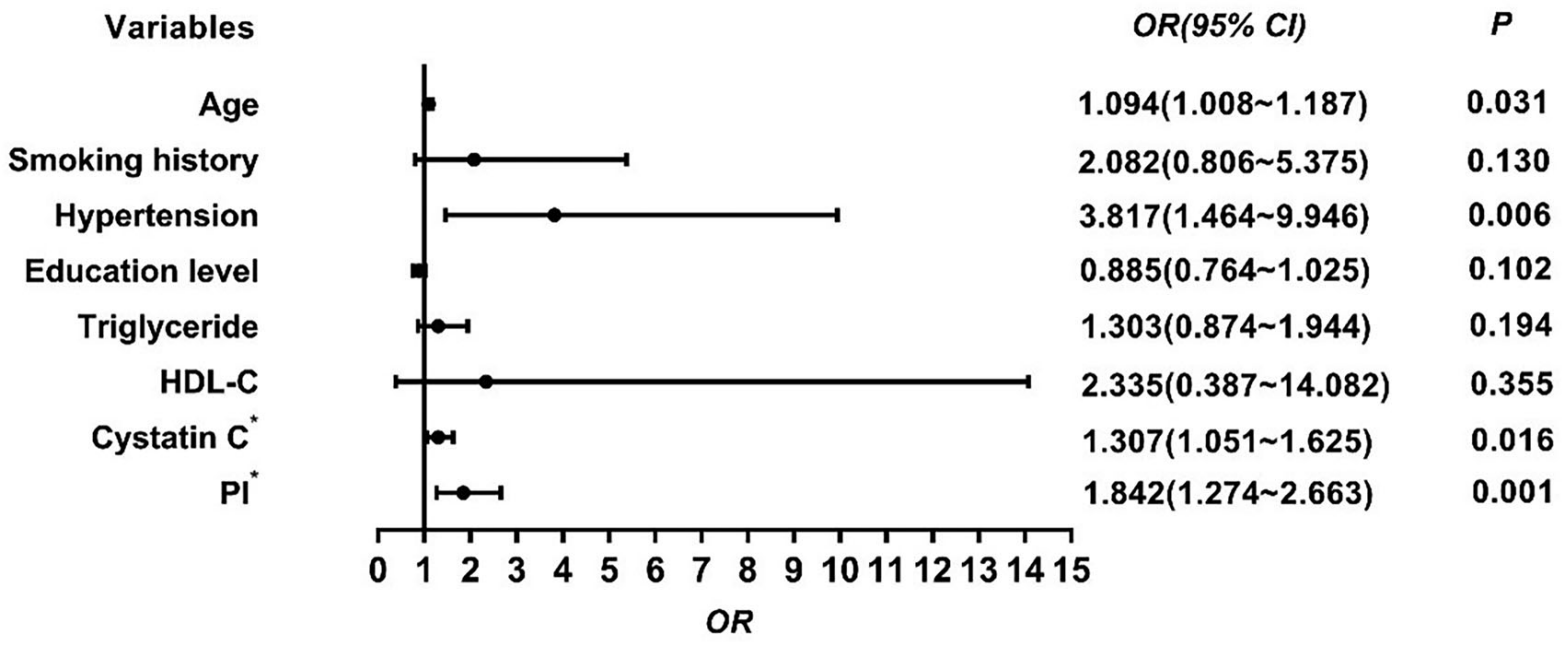
The logistic regression analysis between possible predictors and total cerebral small vessel disease (CSVD) burden. TG, triglyceride; HDL‐C, high‐density lipoprotein; PI, pulsatility index. *, Cystatin C and PI were multiplied by 10 and incorporated into the model.

The results in Table 3 show the high‐burden group had a higher PI level than the low‐burden group (*p *< .001). Factors showing significant differences were examined by multivariate binary logistic regression analysis. After adjusting for potential confounding factors, including age, hypertension, smoking history, education, triglyceride, and cystatin C, PI remained a significant independent risk factor (*p *< .001) for a high burden of CSVD (Figure 2). Spearman correlation analysis found a positive correlation between PI and total burden score (*r* = .392, *p* < .001).

### 3.5 Predictive value of combined PI and total CSVD burden for cognitive impairment

**FIGURE 3:**
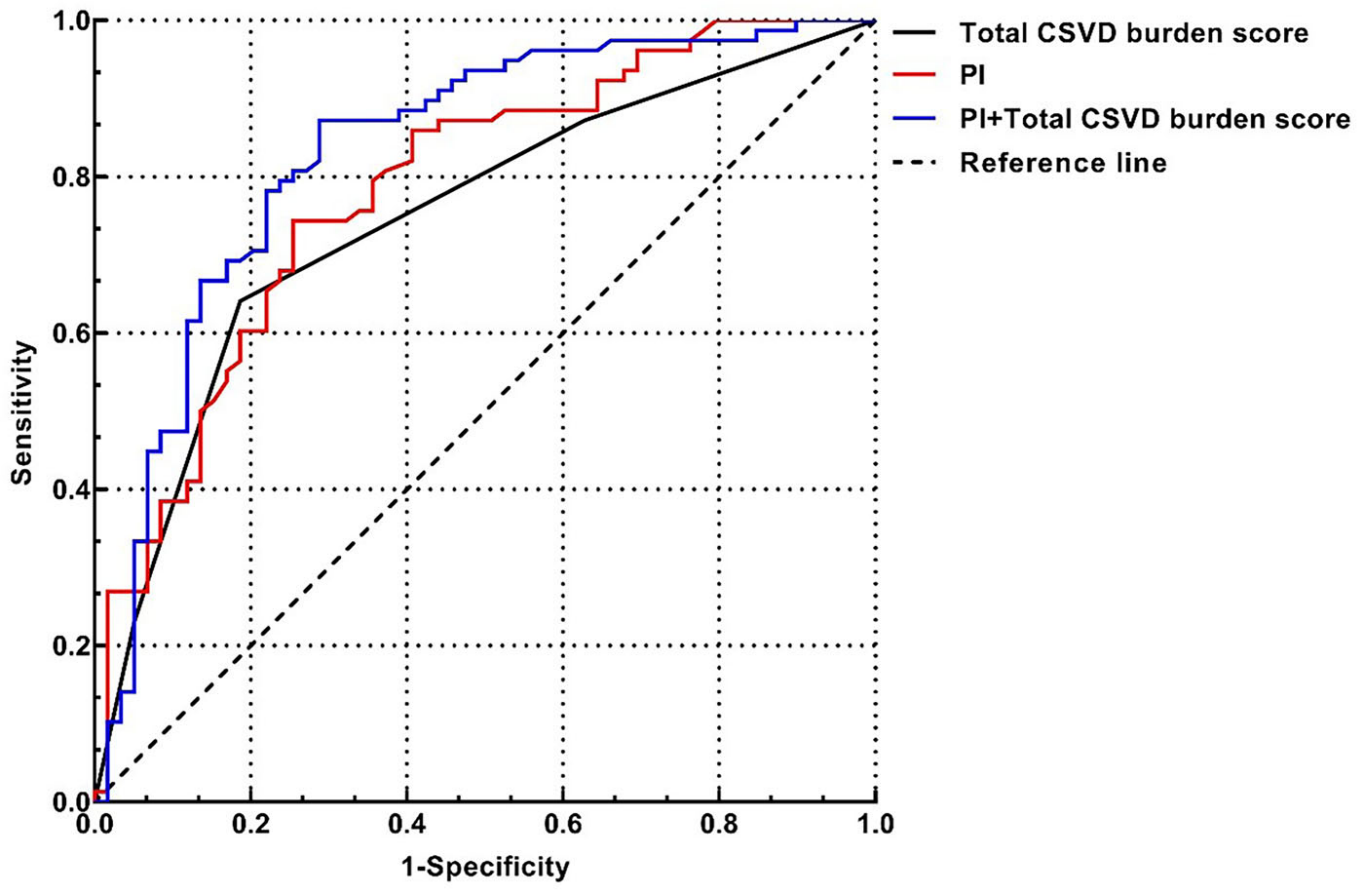
Receiver operating characteristic (ROC) curves for the predictive value of pulsatility index (PI) and total cerebral small vessel disease (CSVD) burden score for cognitive impairment.

The optimal cut‐off value for PI and the area under the curve (AUC) for predicting cognitive function were 0.936 and 0.784 (Table 4). Similarly, the optimal cut‐off for the total burden of CSVD and the AUC for predicting cognitive function were 2.5 and 0.747 (Table 4). The combination of PI and total CSVD burden showed a better predictive value for CI (AUC = 0.832) than the two alone (Figure 3).

**TABLE 4:** Receiver operating characteristic (ROC) curve analysis of pulsatility index (PI) and total cerebral small vessel disease (CSVD) burden score for cognitive impairment.

Index	PI	Total burden score	PI + Total CSVD burden score
AUC	0.784	0.747	0.832
95% CI	0.707–0.861	0.664–0.830	0.761–0.904
*p*	<.001	<.001	<.001
Cut‐off value	0.936	2.5	0.401
Sensitivity	74.4	64.1	87.2
Specificity	74.6	81.4	71.2

## 4 DISCUSSION

The present study investigated patients with CSVD for associations between PI and total CSVD burden with CI. CSVD patients with CI showed significantly higher PI levels than those without CI. Furthermore, PI was identified as an independent risk factor for both CI and high CSVD burden. A significant negative correlation was observed between PI and cognitive function, and a significant positive correlation was observed between PI and total CSVD burden. Notably, the predictive value for CI was enhanced when PI was combined with the total CSVD burden, as opposed to PI alone.

Previous authors have also investigated the correlation between elevated PI and cognitive decline. A longitudinal study spanning 6 years reported a notable association between elevated PI in the middle cerebral artery and CI, thereby increasing the likelihood of dementia and suggestive of early stage Alzheimer's disease (Chung et al., 2017). Furthermore, a cross‐sectional study highlighted that augmented PI signifies insufficient cerebral perfusion and heightened vascular resistance, thereby offering a basis for predicting future risk of dementia and executive dysfunction in CSVD (Vinciguerra et al., 2019). Previous research has indicated that an elevated PI indicates heightened distal vascular resistance, microvascular injury, and compromised function, ultimately resulting in tissue damage and cognitive decline (Cooper et al., 2016; Mitchell et al., 2011; Webb et al., 2012). Several authors reported strong correlations between increased aortic stiffness and pulsatility, and the onset of CI (Cooper et al., 2016; Tomek et al., 2014; Tsao et al., 2016). Memory loss, decreased processing speed, and impaired executive functions have been associated with PI. It is plausible that microvascular damage and remodeling are the underlying mechanism linking aortic atherosclerosis, brain injury, and CI (Mitchell et al., 2011). Here, we found that PI was significantly and negatively correlated with CI and was an independent risk factor for cognitive decline. These findings are consistent with those of previous reports.

Previous workers reported that PI was related to various CSVD imaging markers, thereby increasing the risk of CI. In a study involving individuals with lacunar infarcts, an increased PI may be associated with impairment in several cognitive domains (Altmann et al., 2016). Additionally, a significant correlation between PI and the severity of WML was found in a community‐based study (Mok et al., 2012), but not in patients with lacunar infarcts and CMBs. A systematic review found (Shi et al., 2018) that PI remained significantly correlated with WML and lacunar infarcts even after adjusting for confounders. However, in a prospective study, it was found that patients with severe WML at baseline had significantly elevated PI (*p* < .001) (Kneihsl et al., 2020), which disappeared after adjusting for potential confounders, and PI was not found to be an effective predictor of WML progression after 5 years of follow‐up. Total imaging burden reflects the overall severity of brain injury, and this study revealed positive association between the PI and the total CSVD burden. Importantly, this correlation was still significant following adjustment for possible confounding factors, thereby establishing PI as an independent risk factor for overall CSVD burden. Men and women both have an increase in arterial stiffness with age (Mitchell et al., 2004); women lose the protective effect of estrogen after menopause, and the rapidity of arterial stiffness is more rapid and compliance decreases (Zaydun et al., 2006). Compared to males of the same age, females had a higher increase in wave reflection and pulse pressure; this may be related to women's height and small aortic size (Dart et al., 2008).

Atherosclerosis contributes to heightened aortic pulsation, which compromises the protective Windkessel effect, resulting in elevated systemic arterial pressures (Windkessel effect: Interpretation of the shape of arterial blood pressure waveforms based on interactions between per‐beat output, the compliance of the aorta and elastic large arteries, and the resistance of smaller arteries and arterioles. Large elastic arteries dilate during systole and constrict during diastole. Blood enters the elastic artery through peripheral resistance more quickly than it exits, leaving the aorta with a net reserve of blood during systole that drains during diastole). Consequently, this exacerbates damage to small vessels, induces endothelial dysfunction, and disrupts the blood‐brain barrier (Shi et al., 2018). The brain's autoregulation is impaired in cerebral atherosclerosis, and high pulses lead to wall damage in small intracerebral arteries, promoting the onset and progression of CSVD (Purkayastha et al., 2014; Shi et al., 2018; Webb et al., 2012). Cerebrovascular pulsatility is an important driver of lymphatic system exchange in the perivascular space of the cerebrospinal fluid–interstitial fluid (Iliff et al., 2013; Mestre et al., 2017). It is essential for removing metabolites and other wastes from the brain, and may be one of the potential mechanisms for CSVD to develop.

The development of cognitive impairment may be related to endothelial dysfunction, blood–brain barrier disruption, and inadequate perfusion (Hamilton et al., 2021; Rajeev et al., 2022), similar to CSVD. A case‐control study demonstrated that a high burden of CSVD was an independent risk factor for cognitive decline, and a higher overall burden of CSVD was associated with cognitive dysfunction in middle‐aged and older individuals (Li et al., 2021). Cross‐sectional studies reported that total CSVD burden was linked to overall CI and attention decline (Hosoya et al., 2023), and it was noted that reducing CSVD burden could help to prevent cognitive decline. Inadequate cerebral perfusion, measured by TCD, was linked to cognitive decline and white matter lesions in patients with vascular CI (Malojcic et al., 2017). This was mainly associated with microangiopathy, which results in atherosclerosis, narrowing of vessel diameters, and decreased cerebral blood flow (Malojcic et al., 2017; Sabayan et al., 2012; Vicenzini et al., 2007). Vascular sclerosis and chronic under‐perfusion can lead to disruption of the blood–brain barrier (Yang et al., 2017), ultimately manifesting as cognitive and sensorimotor decline and causing cognitive dysfunction (Marshall et al., 2012; Zachariou et al., 2024). Our study revealed a noteworthy association between PI and CSVD total burden and CI, and the predictive value of PI combined with CSVD total burden for CI was higher than that alone; therefore, PI combined with total burden score was clinically used for better prediction of cognitive functioning in CSVD patients.

This study demonstrated several strengths, including the inclusion of a CSVD population, the ease of performing and obtaining TCD measurements of middle cerebral artery PI, and the exploration of the association between PI and CI. Furthermore, the combination of PI and total burden improved predictive value for CI in patients. Nevertheless, there are several limitations to this study. First, its retrospective nature prevented the investigation of causal relationships. Second, the limited sample size may not adequately represent the broader population, warranting the inclusion of a larger and more diverse sample in future research. Third, because the PI of Doppler ultrasonography is measured manually, it may be affected by the operator's angle and the depth of the probe, albeit by experienced and specialized technicians. Finally, the MMSE scale does not fully reflect some specific cognitive domains, and more cognitive screening tools are needed. In future studies, an increased number of patients will be incorporated, and a greater number of cognitive function assessment scales should be applied to assess the relationship between the middle cerebral artery PI and cognition function.

## 5 CONCLUSION

This study showed that middle cerebral artery PI was associated with CI and severe imaging burden in patients with CSVD. Middle cerebral artery PI combined with total imaging burden improves the predictive value of cognitive impairment in CSVD patients.

## AUTHOR CONTRIBUTIONS

**Huijuan Wu**: Writing—original draft; conceptualization; formal analysis; methodology. **Liaoyang Xu**: Data curation; investigation. **Xingyongpei Zheng**: Investigation; data curation. **Caihong Gu**: Methodology; supervision. **Xinyu Zhou**: Investigation; funding acquisition; writing—review and editing; formal analysis. **Yong Sun**: Writing—review and editing. **Xiaomin Li**: Writing—review and editing.

## CONFLICT OF INTEREST STATEMENT

The authors declare that they have no competing interests.

### PEER REVIEW

The peer review history for this article is available at https://publons.com/publon/10.1002/brb3.3526.

## Data Availability

The data that support the findings of this study are available from the corresponding author upon reasonable request.
